# Stroke mimics: A case report of cervical spondylosis with intravenous thrombolysis

**DOI:** 10.1111/cns.14434

**Published:** 2023-08-31

**Authors:** Yicong Wang, Hui Qu, Wanliang Du, Yilong Wang

**Affiliations:** ^1^ Department of Neurology, Beijing Tiantan Hospital Capital Medical University Beijing China; ^2^ China National Clinical Research Center for Neurological Diseases Beijing China; ^3^ Chinese Institute for Brain Research Beijing China; ^4^ Beijing Key Laboratory of Translational Medicine for Cerebrovascular Disease Beijing China

1

1

Dear Editor,

Stroke, recognized as a leading cause of the global disease burden, has become an increasingly significant public health concern.^1^, ^2^ Nevertheless, it is crucial to differentiate stroke mimics, conditions that exhibit neurological symptoms similar to stroke, particularly in emergency room settings. A comprehensive study involving 8187 emergency patients revealed that approximately 30% of these patients were ultimately diagnosed as stroke mimics.^3,4^ More interestingly, some patients diagnosed with ischemic stroke with intravenous thrombolysis may also end up with stroke mimics.^5^ Here, we report a case of a young male patient who was treated with ischemic stroke with intravenous thrombolysis due to cervical spinal cord compression to improve the recognition ability of stroke mimics.

## 2 CASE REPORT

A 39‐year‐old male patient woke up 6 h ago with neck discomfort and right upper limb pain. He experienced weakness in the right limb, particularly an inability to lift and move the lower limb. There were no issues with consciousness speech, binocular vision, nausea, vomiting, dizziness, difficulty breathing, and nor any history of significant head or neck trauma. Seeking immediate medical attention at another hospital, the patient's vital signs were stable, and his mental state was clear with normal responses. Physical examination revealed no obvious abnormalities in the heart, lungs, or abdomen. The muscle strength of the right upper limb was level 4, the muscle strength of the lower limb was level 2 with a positive Babinski sign; the muscle strength on the left side was level 5. Doctors initially considered acute ischemic stroke as a possibility. After completing the brain and neck computed tomographic angiography showed no intracranial hemorrhage, no obvious signs of large vessel occlusion, or vascular dissection. With the informed consent of the patient and his family members, the alteplase intravenous thrombolysis treatment was performed, but the patient complained that the treatment did not improve significantly, so he was transferred to our hospital to continue emergency treatment.

**FIGURE 1:**
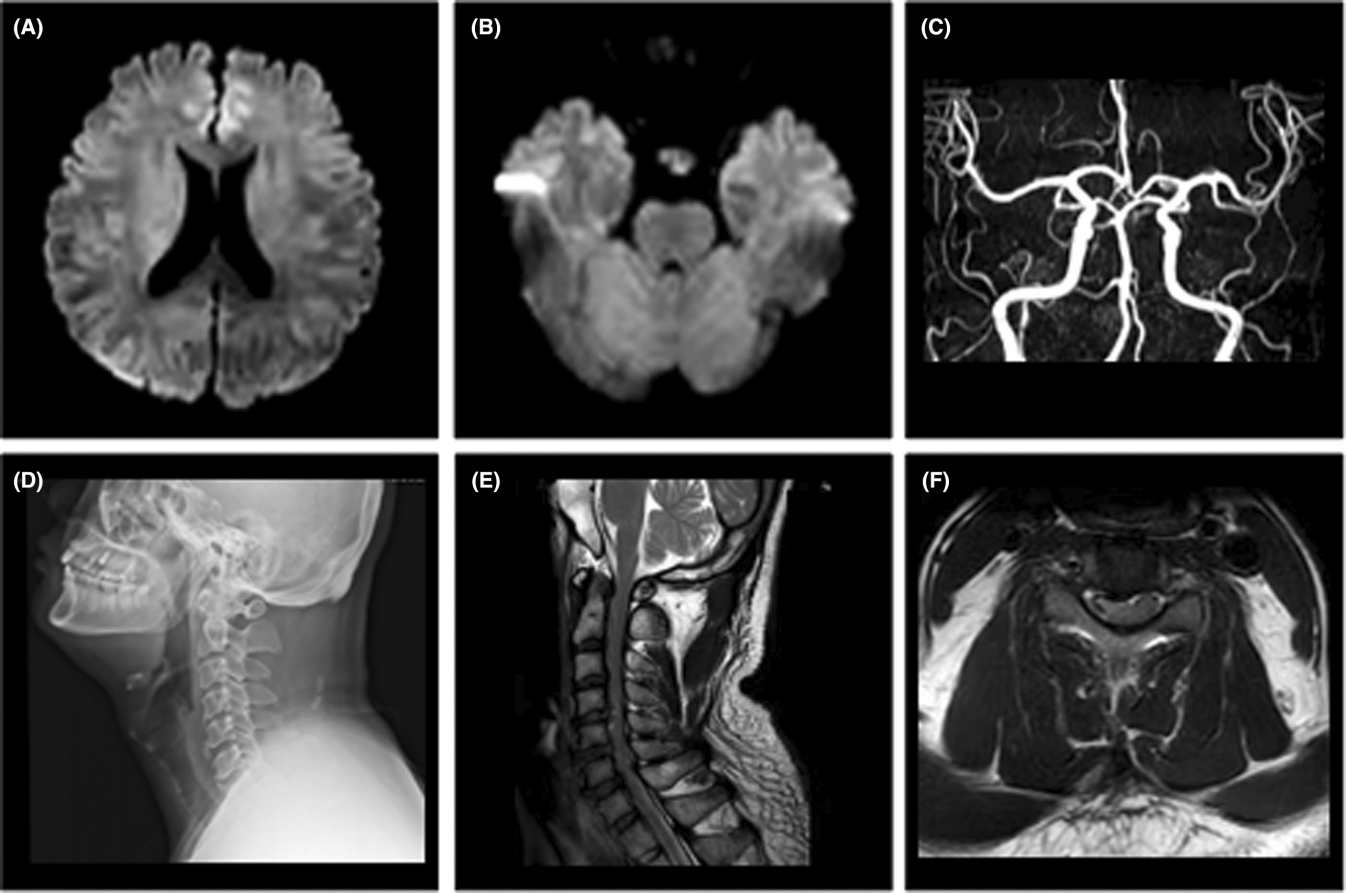
Brain MRI and MRA images and cervical spine X‐ray and MRI. MRI showed no diffusion restriction in the brain (A, B). MRA showed no obvious stenosis or occlusion (C). X‐ray examination revealed that cervical vertebral physiological curvature became straight (D). Cervical spine MRI revealed a patchy T2 high signal that can be seen in the cervical spinal cord at the C4/5 level in both sagittal and axial positions with intervertebral disc prolapse (E, F).

Upon a thorough medical history investigation, it was discovered that the patient worked in an office and had a history of occasional neck pain. He used a massage chair 1 day prior without discomfort. Additionally, he suffered from diabetes for about 3 years, and he has an uncertain history of ankylosing spondylitis. The supplementary physical showed the hypoalgesia on the left below the sternal angle plane compared to the right side. It was found that there was no diffusion restriction in diffusion‐weighted imaging (DWI) or large vessel occlusion in brain magnetic resonance imaging (MRI) or magnetic resonance angiography (Figure 1A–C). Despite receiving intravenous thrombolysis, the comprehensive clinical data did not support a diagnosis of acute ischemic stroke. Further investigations using cervical spine X‐ray and MRI (Figure 1D–F) indicated cervical degenerative changes, intervertebral disc prolapse at C3/4, C4/5, C5/6, and C6/7, and a high signal in the C4/5 cervical spinal cord. Based on these findings, a diagnosis of cervical spondylosis myelopathy was considered, and surgical indications were recognized. The diagnosis of cervical spondylotic myelopathy is considered with surgical indications, the patient was admitted to the hospital for surgical treatment and could walk on your own during follow‐up after 1 month.

## 3 DISCUSSION

Stroke mimics means that they are similar to stroke with neurological deficits but ultimately confirmed as a clinical syndrome of non‐stroke diseases such as epilepsy, migraine, peripheral neuropathy, and so on, which can be developed by intravenous thrombolysis.^6^, ^7^ In cases where patients experience acute onset neurological deficits and exhibit traditional vascular risk factors like diabetes, arriving at a definitive diagnosis based solely on medical history can be challenging. It is difficult to make a clear diagnosis on medical history. However, he was young and had clear neck discomfort and upper limb pain at the early stage, followed by inconsistent weakness in the upper and lower limbs. Cervical spondylosis, particularly when it affects myelopathy, can lead to subtle changes in daily life, such as unstable walking, neck discomfort, radiating numbness, and pain.^8^ When evaluating stroke mimics, obtaining targeted information during consultations becomes crucial. For the diagnosis of dissection, medical history inquiry, massage, trauma, and other triggers can be used to measure bilateral blood pressure, except for evaluating blood vessel imaging. Hence, a comprehensive approach that involves collecting relevant medical history, conducting key physical examinations, and swiftly performing imaging examinations will significantly aid in identifying stroke mimics, especially in young patients.

## 4 AUTHOR CONTRIBUTIONS

All of the authors contributed to this manuscript. Y.C.W. and Y.L.W. contributed to the conception and design; H.Q. and W.L.D. contributed to the acquisition of information; Y.C.W. and Y.L.W. contributed to drafting the text.

## 5 FUNDING INFORMATION

This study was supported by grants from the National Natural Science Foundation of China (No. 81825007), Beijing Outstanding Young Scientist Program (No. BJJWZYJH01201910025030), Youth Beijing Scholar Program (No. 010), Beijing Talent Project ‐ Class A: Innovation and Development (No. 2018A12), “National Ten‐Thousand Talent Plan”‐ Leadership of Scientific and Technological Innovation.

## 6 CONFLICT OF INTEREST STATEMENT

The authors declare that they have no conflict of interest.

## Data Availability

The data that support the findings of this study are available on request from the corresponding author. The data are not publicly available due to privacy or ethical restrictions.
